# Multi-Cohort Exploration of Repetitive Element Transcription and DNA Methylation in Human Steatotic Liver Disease

**DOI:** 10.3390/ijms26125494

**Published:** 2025-06-08

**Authors:** Neil A. Youngson, Aikaterini Tourna, Timothy Chalmers, Kelly V. Prates, Josepmaria Argemi, Ramon Bataller, Koroush S. Haghighi, Lindsay E. Wu, Shilpa Chokshi, Peter Starkel, Patrick S. Western, Margaret J. Morris, Stephen M. Riordan

**Affiliations:** 1 School of Biomedical Sciences, UNSW Sydney, Sydney, NSW 2052, Australiakvp.86@hotmail.com (K.V.P.); lindsay.wu@unsw.edu.au (L.E.W.); m.morris@unsw.edu.au (M.J.M.); 2 Roger Williams Institute of Liver Studies, School of Immunology & Microbial Sciences, Faculty of Life Sciences and Medicine, King’s College London, Foundation for Liver Research and King’s College Hospital, London SE5 9NT, UKshilpa.chokshi@plymouth.ac.uk (S.C.); 3 Department of Biotechnology, Genetics, and Cellular Biology, State University of Maringá, Maringá 87020-900, PR, Brazil; 4 Liver Unit, Clinica Universidad de Navarra, 31008 Pamplona, Spain; jargemi@unav.es; 5 DNA and RNA Medicine Program, CIMA Universidad de Navarra, 31008 Pamplona, Spain; 6 Centro de Investigación Medica en Red (CIBER-EHD), 28029 Madrid, Spain; 7 Instituto de Investigación Sanitaria de Navarra (IdISNA), 31008 Pamplona, Spain; 8 Liver Unit, Hospital Clinic, Institut d’Investigacions Biomediques August Pi i Sunyer (IDIBAPS), 08036 Barcelona, Spain; bataller@clinic.cat; 9 Prince of Wales School of Medicine Clinical School, University of New South Wales, Sydney, NSW 2031, Australia; k.haghighi@unsw.edu.au; 10 Peninsula Medical School, Faculty of Health, University of Plymouth, Plymouth PL4 8AA, UK; 11 Department of Hepato-Gastroenterology, Cliniques Universitaires Saint Luc, 1200 Brussels, Belgium; peter.starkel@saintluc.uclouvain.be; 12 Centre for Reproductive Health, Hudson Institute of Medical Research and Department of Molecular and Translational Science, Monash University, Clayton, VIC 3168, Australia; patrick.western@hudson.org.au; 13 Gastrointestinal and Liver Unit, Prince of Wales Hospital, Randwick, NSW 2031, Australia; 14 Faculty of Medicine and Health, UNSW Sydney, Sydney, NSW 2033, Australia

**Keywords:** alcohol-related liver disease (ARLD), colorectal liver metastases (CRLM), DNA methylation, formalin-fixed paraffin-embedded (FFPE) liver biopsy, hepatocellular carcinoma (HCC), long interspersed nuclear element (LINE), metabolic dysfunction-associated steatohepatitis (MASH), metabolic dysfunction-associated steatotic liver disease (MASLD), non-alcoholic fatty liver disease (NAFLD), non-alcoholic fatty liver disease activity score (NAS), steatosis grade (SG), transposable elements (TEs)

## Abstract

Transposable elements (TEs) make up around half of the human genome. Their transcription is repressed in most somatic cells to maintain genome integrity and function. The repression is chiefly maintained by a combination of epigenetic modifications such as DNA methylation and histone modifications. However, recent research suggests that liver steatosis is associated with extensive changes to the hepatocyte epigenome. Furthermore, studies in mice have reported diet- and drug-induced changes to TE transcript levels in liver. The confirmation of these effects in human liver has not previously been undertaken. Here, we examined TE transcription in liver tissue from three patient cohorts with histologically confirmed liver steatosis caused by alcohol consumption or metabolic dysfunction. The quantitation of the number of transcripts with TE-homology in RNA-Seq data from a cohort of 90 bariatric surgery patients with metabolic dysfunction-associated steatotic liver disease (MASLD) revealed a trend for the reduction in TEs of all classes due to increasing steatosis, but no effect of fibrosis. This pattern was also present in a separate cohort of MASLD and HCC patients, as RT-qPCR also showed a reduction in Alu element transcripts in advanced steatosis, but again, no effect of fibrosis. Contrastingly, in a cohort of alcohol-related liver disease patients, the reduction in LINE-1 transcripts was associated with either increased steatosis or increased fibrosis. Moreover, the examination of LINE-1 DNA methylation levels in the MASLD and HCC cohort indicated that DNA methylation was also negatively associated with LINE-1 transcription in MASLD. This study suggests that TE transcript levels in human liver are slightly reduced by steatosis, that DNA methylation is an influential epigenetic regulator of LINE-1 retrotransposon transcription in steatosis, and that Alu transcript levels in background liver could be a new biomarker for HCC in cirrhotic and non-cirrhotic MASLD.

## 1. Introduction

Between 45% [1] and 69% [2] of the mammalian genome is repetitive DNA, predominantly consisting of sequences termed transposable elements (TEs) [1]. While many of these sequences have lost their ability to reproduce since their initial integration, the abundance of these sequences is due to their replicative ability, mainly through the integration of DNA from reverse-transcribed RNA (retrotransposition), which has led to their accumulation over hundreds of thousands of years. The two most abundant types of TEs in human and mouse are Long and Short Interspersed Nuclear Elements (LINEs/SINEs). Repetitive DNA can disrupt gene function by increasing the risk of DNA replication errors such as those leading to genome rearrangements, or the retrotransposition of TEs into regions that alter that gene coding sequence or regulation [1,3]. To counter these potentially deleterious events, repetitive DNA is usually epigenetically repressed, typically by high levels of DNA methylation, repressive histone modifications, and low levels of transcriptionally permissive histone acetylation, which reduce transcription and contribute to the maintenance of genome integrity [1,3]. Despite the genome-wide repressive epigenetic state of most repetitive DNA, low levels of transcripts from TEs have been detected in all tissues [4]. It is currently unclear whether this low-level transcription is harmful to the cell, is normal, or is beneficial, as there are examples of cell-specific functions of TE transcripts in preimplantation development, brain development and function, and cellular stress responses [1,3].

There is abundant evidence for epigenetic changes in liver disease at specific genes, as well as genome-wide or ‘global’ changes [5,6,7,8]. In fatty liver diseases associated with alcohol consumption or metabolic dysfunction, these changes can be part of the transcriptional responses required to metabolise excess ethanol, lipids, or carbohydrates, or responses to pathological processes such as lipotoxicity, inflammation, and oxidative stress. However, as well as these ‘transcriptional response’ epigenetic changes, there is evidence that epigenetic changes in hepatocytes can be induced through perturbation of the epigenetic remodelling mechanisms themselves. This is due to the highly intertwined pathways of liver metabolism and epigenetics [9]. Hepatocytes are highly metabolically active through the functions of processing dietary carbohydrates and lipids, detoxification of xenobiotics, and production of bile acids. Intermediary metabolites of all three of these processes [10,11,12] have been discovered to either be the substrates for epigenetic modifications, or to regulate the function of the enzymes which ‘write’ or ‘erase’ the modifications. Two key metabolism-derived epigenetic mechanisms with high activity in hepatocytes are one-carbon metabolism, which generates S-Adenosyl methionine (SAM), the substrate for DNA and histone methylation, and the production of the histone acetylation substrate, acetyl-CoA, from the catabolism of lipids and carbohydrates [10]. The perturbation of one-carbon metabolism due to low methionine diets [13] or an increased acetyl-CoA flux from high energy diets [5,14] in rodent models have been shown to reduce genome-wide methylation (of DNA and histones) and increase histone acetylation, respectively, in the liver. In the case of low methionine diets, the reduced genome-wide DNA and histone methylation are associated with the increased transcription of TEs, presumably due to a release from their repression [15]. Furthermore, beyond these extreme physiological impacts on hepatocyte states, recent evidence in rodent studies has shown that moderate exposures to drugs, and various dietary macronutrients, can increase or decrease TE transcripts [16] and DNA methylation [17] in the liver, suggesting that TE transcription in hepatocytes is highly dynamic and sensitive to environmental exposures. However, less is known about how diet and drug exposures in humans affect liver TE epigenetic states and transcription.

In this study, we investigated whether TE transcript levels were altered in patients with metabolic dysfunction-associated steatotic liver disease (MASLD), alcohol-related liver disease (ARLD), and MASLD-associated hepatocellular carcinoma (HCC). MASLD, previously known as non-alcoholic fatty liver disease (NAFLD), or metabolic dysfunction- associated fatty liver disease (MAFLD), is a common condition, affecting around 15–25% of the world’s population, with continued increases in prevalence projected as part of the general increases in metabolic disease [18]. MASLD, like ARLD, is a chronic condition which initiates with elevated lipid storage (simple steatosis). The liver can proceed to steatohepatitis, either metabolic dysfunction-associated steatohepatitis (MASH), or alcoholic steatohepatitis (ASH), through increasing inflammation and fibrosis and eventually cirrhosis and liver failure [19,20]. Both MASLD and ARLD increase the risk for the development of HCC [19]. While several studies have identified epigenetic changes at genes in MASLD [21,22,23,24] and ARLD [8], much less is known about how repetitive elements are affected in these human diseases. TE retrotransposition has been shown to be able to create driver mutations in HCC in some advanced ARLD patients [25], but the frequency of this and the role of epigenetics are unclear. One recent study suggests that similar genome-wide epigenetic dysregulation occurs in humans as in rodent models of fatty liver. Lai et al. reported reduced DNA methylation in MASLD and overweight groups compared to a control group with lower body weight [26]. As altered TE transcript levels could contribute to liver disease pathology, or even be revealed to have a hepatocyte-specific function, it is important to uncover whether TE levels and epigenetic states are also dynamic and influenced by steatohepatitis in humans. This multi-disease investigation (MASLD and ARLD) necessitated a multi-centre and multi-cohort approach, and an inevitable trade-off between the breadth of diseases covered versus the ability to control for some study variables. However, as this is the first study dedicated to the examination of TEs in human liver steatosis, we wished to examine both ARLD and MASLD to create a broad foundation for this research.

## 2. Results

### 2.1. Examination of Transposable Element Transcripts in RNA-Seq from Pre-Cirrhotic Bariatric Surgery Patients (Cohort 1)

To examine the relationship between MASLD and TE transcription, we quantified the bulk RNA-Seq sequence reads with homology to TEs in a well-characterised cohort of liver samples, previously used by Pantano and colleagues [27]. The liver samples were from patients undergoing bariatric surgery and had the severity of disease histopathologically determined. A feature of that cohort, which was attractive for our study, was that Pantano et al. [27] utilised single-cell transcriptome information to characterise the shifts in liver cell composition through the disease spectrum. We used TEtranscripts [28] to measure TE expression at the family level in 90 samples, which represented control/healthy livers or the pre-cirrhotic stages of MASLD. The demographic data of the cohort are presented in Supplementary Table S1. Body Mass Index (BMI) data were not available for each individual patient RNA-Seq file; however, all groups (including the NAS 0 fibrosis stage 0 groups) had an average BMI > 40 due to all being bariatric surgery patients [27]. We focussed on the pre-cirrhosis stages of MASLD to limit the influence of cellular composition differences in the liver, which were evident from fibrosis stage 2 onwards in the cohort, when macrophages and cholangiocytes proportionally increase with a corresponding reduction in hepatocytes [27]. Therefore, at these early stages of MASLD, changes in gene and TE transcript levels can more confidently be attributed to changes in the hepatocyte state rather than due to changes in the cellular composition of the tissue samples. Importantly, even within this reduced spectrum of disease, the 90 liver samples displayed a range of steatosis and fibrosis, which allowed us to evaluate whether these two major aspects of MASLD pathology are associated with changes to TE transcript levels.

**Figure 1:**
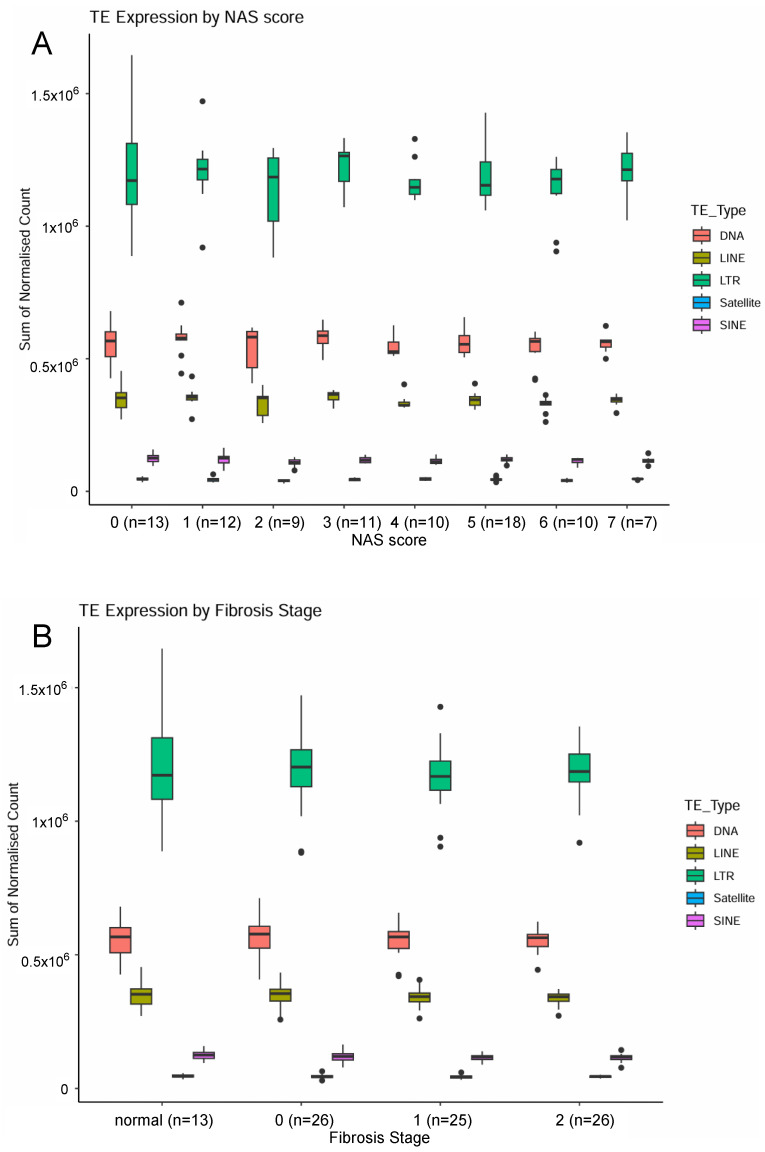
Quantification of RNA-Seq reads with homology to repetitive element subclasses in pre-cirrhotic MASLD human liver. Samples from 90 male and female samples classified by NAS score (steatosis) (**A**) or fibrosis (**B**). Normalised repeat counts presented as mean ± SD. DNA, DNA transposons; LINE, Long Interspersed Nuclear Elements; LTR, Long Terminal Repeat retrotransposons; Satellite, Satellite repeats; SINE, Short Interspersed Nuclear Elements.

TE transcript levels were compared at the classification levels of all TEs, subclass, and family, with clinical samples categorised by the level of steatosis (NAS score) or fibrosis stage. When examining all reads with homology to TEs (Supplementary Figure S1), we observed a non-significant trend for reduction as the NAS score increased (NAS 0–3 vs. NAS 4–6, all fibrosis 0–1, *p* = 0.08 Mann–Whitney U test), but less influence of fibrosis in the same samples (Fibrosis normal/0 vs. 1, *p* = 0.33 Mann–Whitney U test). The same non-significant pattern was seen in all common subclasses of TE (Figure 1). To identify if any TE-regulating gene or pathway could explain the trend for the reduction as the NAS score increased, the differential expression analysis of genes was performed with the same samples (NAS 0–3 vs. NAS 4–6, all fibrosis 0–1). The Gene Ontology analysis of all *p* < 0.05 genes revealed that the most significant KEGG Biological Processes enriched with genes that had higher levels in NAS 4–6 were inflammation and innate immunity-related, while the genes that had lower levels in NAS 4–6 were enriched for lipid, steroid, and one-carbon metabolism processes (Supplementary File S1). The examination of candidate TE-regulatory genes revealed significantly higher transcript levels in high NAS for *APOBEC3G*, *RB1*, *SAMHD1*, and *SUV39H2*, and significantly lower transcript levels in high NAS for *MAT1A* (all *p* = 0.02 to 0.03). However, no gene remained significant after adjustment for multiple testing (Supplementary Table S2).

The examination of TE transcripts at the family level did identify some which were significantly increased or decreased by fibrosis or steatosis (Table 1, Supplementary Figure S2). However, the significantly affected TE families were not limited to any particular subclass.

### 2.2. Examination of LINE and SINE Transcripts and DNA Methylation in FFPE Samples from Chronic Liver Disease and HCC Patients (Cohort 2)

To complement our genome-wide study on moderate liver disease in bariatric surgery patients, we investigated TE transcripts in a separate cohort of broader disease severity. This cohort was also investigated with different methodologies. We performed RT-qPCR on Alu and LINE-1 (L1) elements in RNA extracted from ex-diagnostic Formalin-Fixed Paraffin-Embedded (FFPE) liver biopsy or liver resection samples from ‘Normal’ livers (non-steatotic background liver from resections performed on Colorectal Liver Metastases (CRLM) patients), as well as simple steatosis, MASH/cirrhosis, and HCC samples from patients with a MASLD aetiology. In addition, we measured L1 element methylation in DNA from the same samples to evaluate the role of this epigenetic modification in TE transcription at various stages of the disease. The histopathological staging of the FFPE samples allowed us to stratify data by steatosis grade, NAS, and fibrosis stage, to evaluate the effects of staging on TE transcription and DNA methylation, and to allow for a comparison with the first cohort.

We chose to focus on LINE-1 and Alu for several reasons. They are the most abundant and most transcriptionally active TEs in the human genome [2,3]. There is also evidence for their retrotransposition causing gene mutations, including as driver mutations in liver cancer [1,3,25]. Additionally, the availability of commercial resources for the targeted methylation analysis of LINE-1 allowed a direct comparison of transcription and DNA methylation in the FFPE samples of cohort 2.

The demographic data of the study cohort are presented in Supplementary Table S3. They reveal that the age and gender proportions were similar between normal and MASLD groups, although the MASLD-associated HCC group had a higher proportion of older males. The distributions of steatosis grades, non-alcoholic fatty liver activity scores (NAS) and fibrosis stages in 45 patients with MASLD are presented in Supplementary Table S4. As expected, there was a positive correlation between fibrosis score and NAS (Supplementary Table S5). No overall effects of sex were seen on the three parameters studied in this cohort—L1 and Alu transcript levels, and L1 methylation (Supplementary Table S6).

As for the first cohort, we examined the effects of steatosis and fibrosis on TE transcript levels. Steatosis was investigated in two ways: with NAS, which examines steatosis, lobular inflammation, and hepatocyte ballooning, and with the steatosis grade. No significant differences were observed in Alu or L1 transcript levels between normal livers, NAS 1–3, or NAS ≥ 5 (Table 2). There was a trend for reduced DNA methylation levels in the NAS ≥ 5 group versus the other two. When the normal and NAS 1–3 groups were combined (52.5 ± 4.5, n = 27), this became significant (*p* = 0.045) when compared to NAS ≥ 5, (34.6 ± 7.0, *n* = 10).

Classification of MASLD samples by steatosis grade identified a significant reduction in Alu transcript levels in grade 3 compared to normal, grade 1, and grade 2 steatosis (Table 3). DNA methylation levels of L1 was also reduced in this group compared to normal and steatosis grade 2 groups.

No significant differences were observed in Alu or L1 transcript levels or DNA methylation levels between normal livers or MASLD samples with fibrosis stages 0–2 or 3–4 (Table 4).

Unlike the bariatric surgery cohort, this cohort allowed for the examination of TE transcription methylation in samples from patients with HCC. Alu transcript levels were significantly lower in non-HCC tissue from patients with HCC than in the liver in patients without HCC (Table 5). This difference was still present when patients with steatosis grade 3 were excluded (second row of Table 5). There was a trend for reduced DNA methylation levels in the same group comparisons.

As well as between-group comparisons, we examined the association between L1 DNA methylation and L1 transcript levels within individual groups. This revealed a consistent negative association, which was significant in the group with all samples, as well as within all MASLD samples (Table 6).

### 2.3. Examination of LINE and SINE Transcripts in RNA Extracted from Fresh Liver Biopsies from ARLD Patients (Cohort 3)

**Figure 2:**
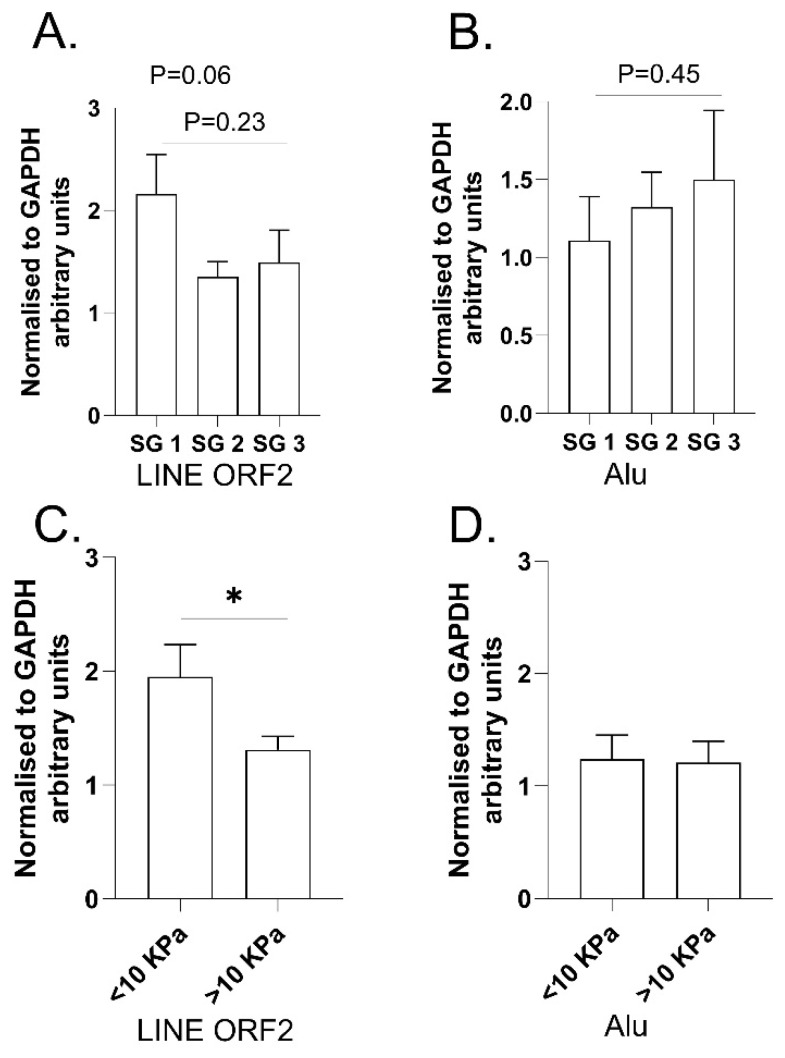
RT-qPCR for L1 and Alu in human ARLD patient liver samples. LINE-1 transcript levels were reduced with increasing (**A**) steatosis severity or (**C**) liver stiffness. Alu transcript levels were unaffected by steatosis severity (**B**) or liver stiffness (**D**). In (**A**,**B**) *n* = 7 steatosis grade (SG)1, *n* = 8 SG2, *n* = 5 SG3 as determined by liver biopsy. In (**C**,**D**), *n* = 11 < 10 KPa, *n* = 14 > 10 KPa. Liver stiffness was determined with Fibroscan^®^. Steatosis was graded by the percentage of hepatocytes with cytoplasmic accumulation of fat droplets as absent, mild (<33% of hepatocytes), moderate (33–66%), or severe (>66%) [20]. The cut-off of +/−10 kPa is similar to a separation of F0-2 versus F3-4 in this patient cohort [29]. Group comparisons done with Student’s *t*-test and presented as mean ± SEM, * *p* = 0.034. L1, LINE ORF2, Long Interspersed Nuclear Element Open Reading Frame 2.

Finally, to investigate whether steatosis or liver fibrosis was associated with TE transcription in ARLD, we performed an RT-qPCR analysis of Alu and L1 repeats in RNA from liver biopsies from alcohol-related liver disease patients. Patient characteristics are presented in Supplementary Table S7. Notably, the average daily alcohol intake of this cohort was 255 g/day compared to under 20 g/day in the MASLD/HCC patients (Cohort 2) and under 30 g/day in the bariatric surgery patients (Cohort 1). The separation of patients into groups based on the level of liver steatosis revealed a trend for L1 transcript reduction in the steatosis grade 2 and steatosis grade 3 samples compared to steatosis grade 1 (Figure 2A). Whereas the separation of patients by the level of liver stiffness revealed a significant reduction of L1 transcripts in patients with higher stiffness (*p* = 0.03) (Figure 2C), no significant differences were seen in these comparisons for Alu elements (all *p* > 0.4) (Figure 2B,D).

## 3. Discussion, Limitations and Conclusions

### 3.1. Discussion

Despite an increase in worldwide research into the pathology and treatment of steatotic liver diseases, and the hepatocellular carcinomas which they promote, the global incidence of these diseases continue to rise [30]. Broader insight into the drivers of these diseases is needed, along with innovative approaches to therapy and patient management. As far as we are aware, our study is the first dedicated to examining TE transcription in steatotic liver disease. This is necessary, as firstly, around half of the human genome is TEs, secondly, as there are recognised genetic and epigenetic contributions to steatotic liver diseases [31], and thirdly, as TE transcription is known to have pathogenic roles in other diseases [1,3]. While repetitive element transcription can influence pathology through many different mechanisms [3], the clearest ones in steatotic liver disease would be to either act locally to influence the expression of nearby genes with known roles in disease (e.g., *PNPLA3*), or to have broader effects on genome function. In cancer cells, local and genome-wide effects of TEs can cause oncogene activation, the repression of tumour suppressor genes, and genome-wide karyotypic changes [32]. In our previous work, we identified that increased lipid deposition in hepatocytes induced genome-wide histone acetylation, which is a transcriptionally permissive modification that can activate repetitive elements in cell culture [5]. Therefore, a logical progression was to examine patient samples to identify whether repetitive element transcription is altered in vivo.

In choosing samples for investigation, we aimed to control as best as possible for the major anthropometric determinants of disease such as age and gender, and for the cellular composition changes that the liver undergoes as disease progresses. In the severe stages of disease, the relative proportions of liver cell types differ drastically from earlier stages [27]. This makes it hard to determine whether an epigenetic or transcriptional change is due to a change specifically within hepatocytes or is due to a relative increase in the proportion of non-hepatocyte cell types which have different repetitive element dynamics. Nonetheless, in samples from patients with early to moderate disease with minor immune infiltration, it is possible to assess whether either of the main pathological processes of steatotic liver diseases, steatosis itself, and fibrosis, can independently alter repetitive element transcription. In this study, we were able to achieve this in three separate patient populations, as steatotic liver disease is a broad term which includes many aetiologies.

There are now a range of studies that provide RNA-Seq data from liver samples of MASLD (or NAFLD) patient cohorts. For cohort 1 of this study, we chose to examine the datasets from Pantano et al., (2021) [27] for several reasons. Firstly, this cohort was collected from a single clinic at the Massachusetts General Hospital (Boston, USA), eliminating the variation introduced by collection from multiple centres. Secondly, the liver biopsy samples used were from a cohort of histologically characterised bariatric surgery patients, with steatosis and fibrosis levels clearly defined. Thirdly, cell type deconvolution was performed to reveal which disease severity level results could be most influenced by shifts in relative cell proportions rather than the hepatocyte state. Finally, a considerable number of RNA-Seq datasets allowed good coverage of a wide variety of pathological stages and all were publicly available from the Gene Expression Omnibus (GEO).

Cohort 2 of this study was a collection of ex-diagnostic FFPE liver biopsy and liver resection samples from the Prince of Wales Hospital Sydney, Australia. Importantly, these patients overlapped pathologically with the patients in the cohort 1 bariatric surgery patients but included MASLD patients who also had HCC.

The ARLD patient samples of cohort 3 were all collected from the Cliniques Universitaires Saint-Luc (Brussels, Belgium) and allowed for the comparison of the effects of steatosis and fibrosis due to alcohol consumption.

Overall, steatosis had a stronger effect on TE transcript levels than fibrosis in the two MASLD cohorts. The trend for reduction across all TE subclasses in samples with a NAS ≥ 4 (Figure 1A and Figure S1A) in bariatric surgery cohort 1 mirrored a significant decrease in Alu transcript levels in samples with a steatosis grade > 3 (Table 3) in the MASLD/HCC cohort 2. Mechanistically, this reduction may be linked to an increase in regulators of repressive heterochromatin (*SUV39H2* [33], *RB1* [34]) or the post-transcriptional degradation of TE transcripts [35] (*APOBEC3G*, *SAMHD1*). The broad range of affected TEs in cohort 1 may suggest that *RB1* is the best candidate [34]. However, as the global reduction in cohort 1 is a trend, further work in other cohorts is required to confirm whether steatosis has more influence on global TE transcript levels than fibrosis. Furthermore, it is likely that there are multiple regulatory mechanisms and/or aetiology-specific factors at play, as L1 transcripts did not have this pattern in the MASLD/HCC cohort 2. The ARLD cohort 3 indicated that LINE-1 transcription was decreased with increasing pathological severity, although both steatosis and fibrosis were associated with this effect. Future biomarker-focused studies could determine whether this change is detectable in blood and is associated with a risk for disease progression.

While ARLD and MASLD have similarities in pathology, such as the initial accumulation of lipids, which can progress through inflammation and fibrosis to cirrhosis, liver failure, and HCC, there are important underlying mechanistic differences which could explain differences in the results [36]. For example, the metabolic processes leading to liver steatosis differ, with increased energy intake being the main driver in MASLD, and the activation of lipogenesis by metabolites of ethanol catabolism being the main driver in ARLD [37].

Our study offers mechanistic insight into TE dynamics in MASLD through the combined examination of L1 transcript levels and L1 DNA methylation in the ex-diagnostic FFPE liver samples of cohort 2. As expected, there was a negative correlation between L1 transcription and DNA methylation [38] when all samples were grouped or separated into normal, MASLD, or HCC (Table 6). This suggests that the transcriptionally repressive effects of DNA methylation are a determinant of L1 transcript levels regardless of the disease state. However, the comparison of the group median levels indicated a more complex relationship across MASLD severity, as well as in HCC patients. L1 methylation was significantly reduced in severe steatosis, as determined by either NAS ≥ 5 or steatosis grade 3 compared to normal or milder MASLD (Table 2 and Table 3), but no effects of fibrosis level were observed (Table 4). Fitting with the expected repressive activity of DNA methylation, L1 transcript levels appeared highest in NAS ≥ 5 or steatosis grade 3 groups, though this did not reach statistical significance. The individual repetitive element analysis of the RNA-Seq (Table 2) data indicated that the CR1 family of L1s may be particularly prone to methylation and transcription changes in higher stage steatosis. This ancient L1 family has also been shown to be hyperacetylated and transcriptionally increased in systemic lupus erythematosus [39].

However, the decrease in Alu transcription in severe steatosis (Grade 3) in Table 3 suggests that not all TEs are affected by large-scale transcriptionally permissive epigenetic changes induced by steatosis (i.e., decreased LINE-1 DNA methylation of this study and increased histone acetylation [5]). Whether the Alu transcript decreases are a cause or consequence of the increasing severity of steatosis in this cohort is unknown, but could be due to the altered regulation of a small group of Alu elements, as they are known to be more affected by local determinants of transcription than other classes of TEs [40].

The reduction in L1 DNA methylation in severe steatosis, whether measured with NAS (Table 2) or steatosis grade (Table 3), may have implications for pathophysiology in steatosis. While not statistically significant, these groups (NAS ≥ 5, steatosis grade 3) did have the highest levels of L1 transcription, which may raise risk for HCC through the insertional mutagenesis of genes [25]. Additionally, demethylated L1 elements could cause the dysregulation of genes through localised actions such as enhancer activity or genetic instability [1] to promote pathologic processes such as lipotoxicity, fibrosis, or cell death. Future work could identify the precise L1 elements which have reduced DNA methylation to investigate their effects on neighbouring genes.

Consistent with previous reports [38,41], in cohort 2, DNA methylation levels in HCC tissue were significantly lower than in the background liver, and correspondingly, the L1 transcript levels trended to be higher (Table 5). However, an intriguing additional effect was uncovered by comparing background liver Alu transcript levels in HCC compared to non-HCC patients. Alu transcript levels were significantly lower in HCC patient background livers than non-HCC livers of a similar steatosis grade, even at steatosis grade 1 and 2 (Table 5). The presence of the difference even at lower steatosis grades is of potential clinical importance, as there are few prognostic biomarkers for HCC in patients without severe liver disease [42]. An Alu level less than or equal to 0.0016 was found in six of seven (85.7%) patients with HCC and in only five of thirty (16.7%) patients without HCC (*p* = 0.0012; Fisher’s exact test). With regard to this cut-off value potentially being considered as a biomarker to clinically predict the presence of HCC, the sensitivity is six out of seven (85.7%), the specificity is twenty-five out of thirty (83.3%), the positive predictive value is six out of eleven (54.5%), and the negative predictive value is twenty-five out of twenty-six (96.2%). These results from our exploratory study encourage larger studies to investigate whether this could be of importance for understanding the origins of carcinogenesis in MASLD-HCC or as a prognostic biomarker. The presence of this latter usage is supported by studies which have used Alu measurements in blood as biomarkers for HCC with cell-free DNA in HCV patients [43], and with cell-free RNA in HBV patients [44].

### 3.2. Limitations

While our study suggests that the genome-wide loss of TE suppression is not a feature of steatotic liver disease progression, a caveat is that we cannot rule out differences existing between non-steatotic livers in healthy individuals and steatotic livers. The ‘Normal’ group in the bariatric surgery cohort 1 all had obesity. In the MASLD/HCC cohort 2, the normal group included non-steatotic background livers from CRLM patients. The ARLD cohort 3 all had high alcohol intake and at least steatosis grade 1. The lack of a healthy control group is partly explained due to liver biopsies being an invasive technique with inherent risks, which make it ethically difficult to justify collection from healthy individuals. However, the possibility for the existence of differences between a healthy liver and steatosis (of any stage) should be noted, particularly considering a report of reduced genome-wide DNA methylation (5-mC levels) in livers even between healthy controls and NAFLD patients [26]. Similarly, cohort 3 had no healthy control or low-ethanol consumption group, again due to the challenges of obtaining liver biopsies from healthy people. Therefore, our study would not detect TE or epigenetic changes caused by ethanol consumption alone. Finally, our genome-wide study cannot rule out single-locus effects on TEs, which could be influential for nearby gene expression. An important extension of this work will be to examine the individual genomic loci of TEs which display expression changes across fatty liver disease severity, to confirm whether the underlying mechanistic changes are transcriptional or post-transcriptional.

### 3.3. Conclusions

We conclude that genome-wide TE activation is not a feature of steatotic liver disease progression due to MASLD or ARLD. In fact, TE repression may be a feature of both. This contrasts with the observed DNA hypomethylation and histone hyperacetylation in steatosis, processes which are both known to activate TE transcription in certain cell types [3,45]. This may indicate that other genome-wide repression mechanisms are at play. Future work should focus on their discovery and whether they affect disease progression.

## 4. Materials and Methods

### 4.1. RNA-Seq Analyses of Bariatric Surgery Cohort 1

In the bariatric surgery cohort 1, liver steatosis and fibrosis were histopathologically determined as described in Pantano et al. (2021) [20,27]. Inclusion and exclusion criteria are also described in that study, with all patients exhibiting a range of MASLD from NAS 0–8 and fibrosis stage 0–4. Other inclusion criteria of note are that all patients had an alcohol intake < 30 g/day (in contrast to cohort 3, below) and patients with non-MASLD causes of chronic liver disease or those with chronic use of steatogenic medications were excluded. However, for our study, we only chose to examine samples with fibrosis stage 0–2, as within those stages, Pantano and colleagues showed with deconvolution analysis that the relative proportions of liver cell types in samples were similar. Any TE transcript changes at ≥F3 could be due to an increased immune cell contribution to the total transcripts.

RNA-Seq datasets were obtained from Gene Expression Omnibus (GEO), deposition number GSE162694. To examine the relationship between MASLD and TE transcription, we quantified the bulk RNA-Seq sequence reads with homology to TEs in a well-characterised cohort of liver samples, previously used by Pantano and colleagues [27]. We downloaded the publicly available raw FASTQ files and associated metadata from NCBI Gene Expression Omnibus using sratoolkit (Version 2.10.8) for 90 samples (Supplementary File S1), representing control/healthy liver or the pre-cirrhotic stages of MALFD. Reads quality was checked and inspected using FastQC (Version 0.11.9). Reads were aligned to the gencode hg38 human reference genome using STAR (Version 2.7.6a) [46], with the following parameters: --outFilterMultimapNmax 100, --winAnchorMultimapNmax 100. Differentially expressed genes between NAS 0–3 and NAS 4–6 (all fibrosis stage ≤ 1) of the cohort 1 datasets were identified with edgeR (v3.36.0 + galaxy 5) [47] on the Galaxy Australia Bioinformatics Platform (https://usegalaxy.org.au/) accessed on 22 May 2025 [48]. Genes with very low expression (counts per million < 1 in a minimum of 3 samples) were excluded. *p* values were adjusted for multiple testing with the Benjamini–Hochberg procedure.

### 4.2. Examination of Ex-Diagnostic Biopsy and Liver Resection FFPE Samples in MASLD/HCC Cohort 2

Ex-diagnostic samples were approved for research by the Prince of Wales Hospital, Sydney, Australia Human Research Ethics Committee, Project Approval HREC ref 17/292 (LNR/17/POWH/585). In this cohort, liver steatosis and fibrosis were histopathologically determined with the same methodology as cohort 1, i.e., the method in Kleiner et al. (2005) [20]. However, steatosis was first analysed just by the level of steatosis (grade 1–3), and secondly with the addition of the other determinants of the NAFLD activity score (NAS), namely lobular inflammation and ballooning. Fibrosis was also staged in accordance with Kleiner et al. (2005) [20].

Exclusion criteria for the cohort were as follows: laboratory testing and obtaining an alcohol intake history excluding an alternative cause for liver disease (and, hence, associated HCC) other than MASLD in all cases. In particular, the testing of peripheral blood for chronic viral hepatitis B and C (hepatitis B surface antigen; hepatitis C virus antibody), autoimmune liver disease (smooth muscle antibody; liver kidney microsomal antibody; antimitochondrial antibody; serum immunoglobulins), alpha-1-antitryspin deficiency (serum alpha-1-antitryspin), haemochromatosis (serum ferritin; serum transferrin saturation), and Wilson’s disease (serum caeruloplasmin; serum copper) proved to be negative. In addition, liver histopathology was in keeping with MASLD and not in keeping with any of these other aetiologies of liver disease in all cases. No patient (including those with liver disease and those with normal liver) gave a history of alcohol intake in excess of 20 g daily; furthermore, all patients had been abstinent from any alcohol intake for at least 6 months prior to inclusion.

DNA and RNA extraction was performed with a Qiagen Allprep for FFPE kit, using the manufacturer’s protocol (Qiagen, Hilden, Germany). RNA was extracted from three 10 μm sections and xylene used for deparaffinization. RNA was treated with DNAse I Amplification Grade (AMPD1-1KT; Sigma-Aldrich, Saint Louis, MO, USA) before being reverse transcribed using the High-Capacity cDNA Reverse Transcription Kit (Applied Biosystems, Foster City, CA, USA), as per the manufacturer’s protocol. Each sample had its own RT control (mastermix without reverse transcriptase enzyme) to allow correction for any genomic DNA contamination after qPCR. Samples were amplified in triplicate with Luna Universal qPCR Master Mix (NEB, Ipswich, MA, USA). Primers which have previously been used to quantify TE transcription were used Alu For 5′-AGTTCGAGACCAGCCTGGC-3′, Alu Rev 5′-CGGGTTCAAGCGATTCTCC-3′ [39,49], which is an Alu-S subclass, hL1-ORF2 F 5′-AAACTGAACAACCTGCTCCTGAATG-3′, hL1-ORF2 R 5′-CTACACACTGCTTTGAATGCGTCC-3′ [50], and normalised to 18S rRNA, with the primers h18S RT F 5′-TGCCCTATCAACTTTCGATGGTAGTC-3′ and h18S 5′-TTGGATGTGGTAGCCGTTTCTCA-3′ [50]. Expression levels were calculated with the delta delta Ct method, with correction for sample-specific gDNA contamination using the RT minus sample.

LINE DNA methylation analysis was performed with a Global DNA methylation LINE-1 kit (Active Motif, Carlsbad, CA, USA, Cat#55017), according to the manufacturer’s instructions. A total of 87 ng–1 μg of genomic was DNA digested with *Mse*I, with the amount determined by the DNA yield from the FFPE extraction. However, after *Mse*I digestion, 100 ng of all samples of DNA was hybridised to the LINE-1 probe. Data are presented as % methylation relative to the methylated and unmethylated kit standards.

### 4.3. RT-qPCR on RNA from Fresh Liver Biopsy in ARLD Cohort 3

Patients with early alcoholic steatohepatitis (ASH) were from Cliniques Universitaires Saint-Luc (Brussels, Belgium), where they were admitted for elective alcohol withdrawal. In this cohort, liver steatosis was histopathologically determined with the same methodology as cohort 1 and 2, i.e., Kleiner et al. (2005) [20]. Fibrosis was determined with transient elastography. All patients were screened with transient elastography and controlled attenuation parameter (CAP) using Fibroscan^®^ at the day of admission. Liver biopsies were performed on the third day after admission via the transjugular approach, when clinically indicated. All patients included gave written informed consent and the research protocols were approved by the institution’s human research and ethical committee (B403201422657).

Inclusion and exclusion criteria for the cohort were as follows. Alcohol intake was self-reported (Supplementary Table S7). The range of alcohol intake in this cohort was 100–750 g/day. All patient samples were analysed together, with no subanalyses of the relatively lower and higher consuming patients. Patients with diabetes were eligible for this study if they were on stable treatment (metformin, oral anti-diabetics with or without insulin) and had a glycosylated haemoglobin level < 7% at admission. Patients were excluded from the analysis if they had one of the following conditions—inflammatory bowel disease or other chronic inflammatory or autoimmune diseases, history of cancer within 5 years prior to admission, known liver disease of any other aetiology, clinically significant cardio-vascular, pulmonary, or renal co-morbidities, clinical and biochemical signs of active infection (temperature > 37.5 °C, viral syndrome, high CRP, and high White Blood cell count), HCV, HBV, or the administration of antibiotics, probiotics, glucocorticoids, and nonsteroidal anti-inflammatory drugs during the two months preceding enrolment.

#### 4.3.1. Fibroscan^®^ Measurements

Transient elastography measurements were performed using the Fibroscan^®^ device (Echosens, Paris, France) by an experienced examiner blinded to the patient’s data. Fibroscan^®^ was considered valid if at least 10 validated measurements were obtained with an interquartile range of less than 30%. The final result, expressed in kPa, was the median of all valid measurements obtained. We used the cut-offs for alcoholic patients proposed by Nguyen-Khac et al. 2018 [51], i.e., 9, 12.1, and 18.6 kPa for F2, F3, and F4, respectively. Significant fibrosis can be assumed based on a cut-off of ±10 kPa.

#### 4.3.2. Transjugular Liver Biopsy and Tissue Sampling

Liver biopsy was performed using a transjugular liver access and biopsy needle (16 G) set (Cook, Bjaeverskov, Denmark). After the administration of local anaesthesia to the neck, a catheter was introduced into the right jugular vein and guided under fluoroscopy into the right main hepatic vein. Separate tissue samples were taken and routinely fixed in formalin, snap-frozen in liquid nitrogen, and preserved in RNAlater solution (Invitrogen, Carlsbad, CA, USA). After a complete routine histopathological examination of the biopsy samples by an experienced liver pathologist, surplus tissue was recovered for further research purposes.

Biopsy samples of ≥15 mm in length, including a minimum of 6 portal tracts, were considered suitable. Steatosis was histologically determined by the percentage of hepatocytes with elevated lipid deposits—mild (<33% of hepatocytes), moderate (33–66%), or severe (>66%) [29].

#### 4.3.3. RT-qPCR Methods

A total of 500 ng of RNA was DNAseI-treated, then reverse transcription, and qPCR undertaken as described above for the MASLD/HCC cohort. cDNA was diluted 1 in 10 with water, and 2 uL of cDNA was used per qPCR.

### 4.4. Statistical Analyses

#### 4.4.1. RNA-Seq Analyses of Bariatric Surgery Cohort 1

TE subfamily expression was measured across samples using TECount from TEtranscripts and the TEToolkit, Version: 2.2.3 [28], with the corresponding count file for each sample merged into a count matrix and imported to R (Version 4.3.1) for differential expression analysis using DESeq2 (Version 1.40.2) [52]. *p* values were adjusted for multiple testing with the Benjamini–Hochberg procedure. Computations for downloading and aligning RNA sequencing files in this project used the computational cluster Katana, supported by Research Technology Services at UNSW Sydney.

#### 4.4.2. Examination of Ex-Diagnostic Biopsy and Liver Resection FFPE Samples in MASLD/HCC Cohort 2

Results were expressed as mean +- SEM or as median and range, depending on whether data conformed to a parametric or non-parametric distribution, respectively. Correlations were determined using Spearman’s rank sum test. When two groups were compared, the unpaired *t* test or Mann–Whitney test were employed, depending on whether data conformed to a parametric or non-parametric distribution, respectively. When three groups were compared, the one-way analysis of variance with Tukey’s test for multiplicity-adjusted *p* values was employed when data conformed to a parametric distribution, while the Kruskal–Wallis test with Dunn’s test for multiplicity-adjusted *p* values was employed when data conformed to a non-parametric distribution. Statistical analyses were performed using GraphPad Prism version 10.0.0 for Windows, GraphPad Software, Boston, MA, USA, www.graphpad.com.

#### 4.4.3. RT-qPCR on RNA from Fresh Liver Biopsy in ARLD Cohort 3

Results were expressed as mean + SEM and groups were compared with the Student’s *t*-test.

## Figures and Tables

**Table 1:** Individual repetitive element families with significant changes due to fibrosis or steatosis. Positive fold change is higher transcript level in 2nd group. lfcSE, log2 fold change standard error; padj, *p* value adjusted for multiple testing. Positive fold change is higher transcript level in 2nd group.

	log2 Fold Change	lfcSE	padj
**Fibrosis normal vs. 0**			
MIRc:MIR:SINE	−0.58	0.18	0.03
Charlie22a:hAT-Charlie:DNA	−0.54	0.17	0.03
MER96:hAT-Tip100:DNA	−0.66	0.21	0.03
**Fibrosis normal vs. 1**			
MER50C:ERV1:LTR	−1.06	0.31	0.01
HERV35I-int:ERV1:LTR	−0.63	0.19	0.02
Charlie4:hAT-Charlie:DNA	−0.59	0.19	0.03
MIRc:MIR:SINE	−0.58	0.18	0.03
**NAS low (0–3) vs. NAS high (4–6)**			
CR1-13_AMi:CR1:LINE	1.39	0.39	0.01
MER50B:ERV1:LTR	0.79	0.26	0.03
MLT1C-int:ERVL-MaLR:LTR	0.85	0.28	0.03

**Table 2:** Alu and L1 transcript levels and L1 methylation levels in relation to non-alcoholic fatty liver activity score in patients with MASLD (median, range, or mean ± SEM). ^1^ *p* = 0.07 compared to normal liver. ^2^ *p* = 0.07 compared to non-alcoholic fatty liver activity score (NAS) 1–3. L1, LINE-1, Long Interspersed Nuclear Elements.

Non-Alcoholic Fatty Liver Activity Score (NAS) in MASLD Patients			
	Normal Liver	1–3	≥5
Alu	(*n* = 12) 0.006490 (0–0.023670)	(*n* = 25) 0.007753 (0–0.154509)	(*n* = 12) 0.006865 (0–0.154500)
L1	(*n* = 12) 0.000269 (0–0.003355)	(*n* = 25) 0.000163 (0–0.003606)	(*n* = 12) 0.000298 (0.000014–0.004378)
L1 Methylation	(*n* = 21) 50.6 ± 4.9	(*n* = 6) 58.8 ± 10.9	(*n* = 10) 34.6 ± 7.0 ^1,2^

**Table 3:** Alu and L1 transcript levels and L1 methylation levels in relation to steatosis grade in patients with NALFD (median, range, or mean ± SEM). # *n* = 2; values of 44.3, 52.7. ^1^ *p* = 0.046 compared to normal liver, ^2^ *p* = 0.04 compared to steatosis grade 1, ^3^ *p* = 0.038 compared to steatosis grade 2, ^4^ *p* = 0.04 compared to normal liver, ^5^ *p* = 0.04 compared to steatosis grade 2. L1, LINE-1, Long Interspersed Nuclear Elements.

Steatosis Grade in MASLD Patients				
	Normal Liver	1	2	3
Alu	*n* = 12 0.006490 (0–0.023670)	(*n* = 8) 0.006752 (0–0.107100)	(*n* = 22) 0.006865 (0–0.154500)	(*n* = 7) 0.000702 ^1,2,3^ (0–0.004928)
L1	(*n* = 12) 0.000269 (0–0.003355)	(*n* = 8) 0.000069 (0–0.003606)	(*n* = 22) 0.000195 (0–0.00438)	(*n* = 7) 0.000456 (0–0.001186)
L1 Methylation	(*n* = 21) 50.6 ± 4.9	#	(*n* = 6) 60.1 ± 10.8	(*n* = 8) 30.2 ± 7.9 ^4,5^

**Table 4:** Alu and L1 transcript levels and L1 methylation levels in relation to fibrosis stage in patients with NALFD (median, range). L1, LINE-1, Long Interspersed Nuclear Elements.

Fibrosis Stage in MASLD Patients			
	Normal Liver	0–2	3–4
Alu	(*n* = 12) 0.006490 (0–0.023670)	(*n* = 24) 0.006064 (0–0.069150)	(*n* = 13) 0.004928 (0–0.107100)
L1	(*n* = 12) 0.000269 (0–0.003355)	(*n* = 24) 0.0002072 (0–0.004378)	(*n* = 13) 0.000203 (0.000014–0.003606)
L1 Methylation	(*n* = 21) 43.7 (13.0–96.1)	(*n* = 6) 54.4 (7.6–81.3)	(*n* = 10) 46.3 (8.9–79.5)

**Table 5:** Study parameters in non-HCC liver tissue in MASLD patients with and without HCC (median, range). ^1^ *p* = 0.0036, ^2^ *p* = 0.043, ^3^ *p* = 0.078 vs. non-HCC liver tissue in patients Without HCC. # Three of four patients with steatosis grade 3. ^4^ *p* = 0.06 vs non-HCC liver tissue in patients without HCC. N/A, as this row is a subanalysis of the top row to show the presence of group differences in the top row even at lower steatosis grades. L1, LINE-1, Long Interspersed Nuclear Elements; non-alcoholic fatty liver activity score (NAS).

HCC Presence Reduces Alu Transcript Levels in Background Liver Tissue in MASLD Patients (HCC Tissue from Same Patients in Last Column for Comparison)			
	Liver Tissue in MASLD Patients Without HCC	Liver Tissue in MASLD Patients with HCC	MASLD-Associated HCC Tissue
Alu	(*n* = 30) 0.007846 (0.000115–0.154500)	(*n* = 7) 0.0001524 (0–0.040070) ^1^	(*n* = 11) 0.003860 (0–0.015690) ^4^
Alu (excluding Steatosis Grade 3)	(*n* = 25) 0.007753 (0.000115–0.154500)	(*n* = 5) 0.0001524 (0–0.040070) ^2^	N/A
L1	(*n* = 30) 0.000273 (0–0.004378)	(*n* = 7) 0.000168 (0–0.002724)	(*n* = 12) 0.000282 (0–0.006193)
L1 Methylation	(*n* = 4) 12.4 (8.9–48.2) #	(*n* = 12) 54.2 (7.6–81.3) ^3^	(*n* = 13) 35.6 (6.2–80.6)

**Table 6:** Correlation between L1 transcript levels and L1 methylation levels (Spearman’s rank correlation test). L1, LINE-1, Long Interspersed Nuclear Elements.

	r	*p*
Overall (*n* = 28)	−0.62	**0.0004**
Normal (*n* = 7)	−0.56	0.21
MASLD (*n* = 10)	−0.78	**0.01**
HCC (*n* = 11)	−0.52	0.11

## Data Availability

The raw data supporting the conclusions of this article will be made available by the authors on request.

## Supplementary Materials

The following supporting information can be downloaded at: https://www.mdpi.com/article/10.3390/ijms26125494/s1.
