# Supplementary material for: Multi-Cohort Exploration of Repetitive Element Transcription and DNA Methylation in Human Steatotic Liver Disease
Source: Int J Mol Sci. 2025 Jun 8;26(12):5494. doi: 10.3390/ijms26125494 (PMC12192986; doi:10.3390/ijms26125494)
Supplement: Supplementary file 1 [file ijms-26-05494-s001.zip › Supplementary Figures and Tables.pdf]

Supplementary Figures

A.

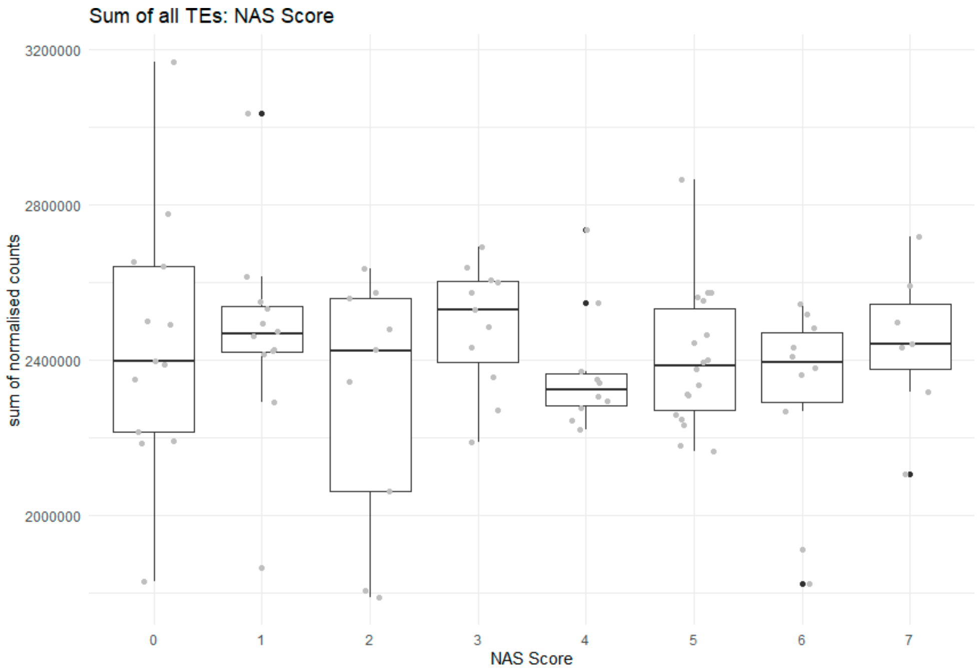

B.

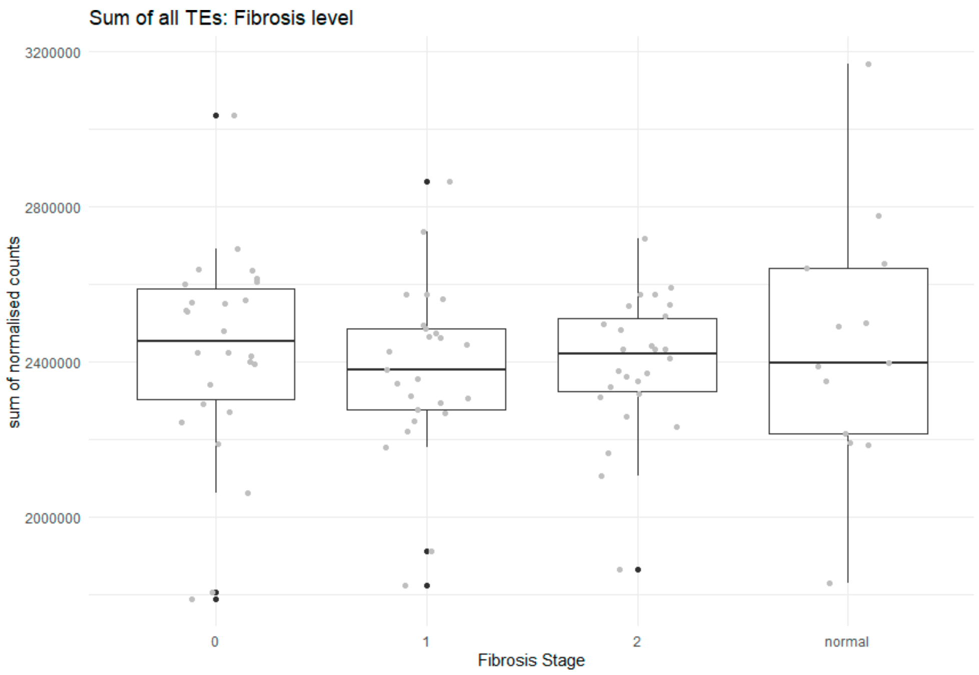

Supplementary Figure S1. Total normalised liver biopsy RNA-seq reads with homology to transposable elements in patient groups from the Cohort 1 bariatric surgery patients. A patients grouped by NAS, B patients grouped by fibrosis.

A.

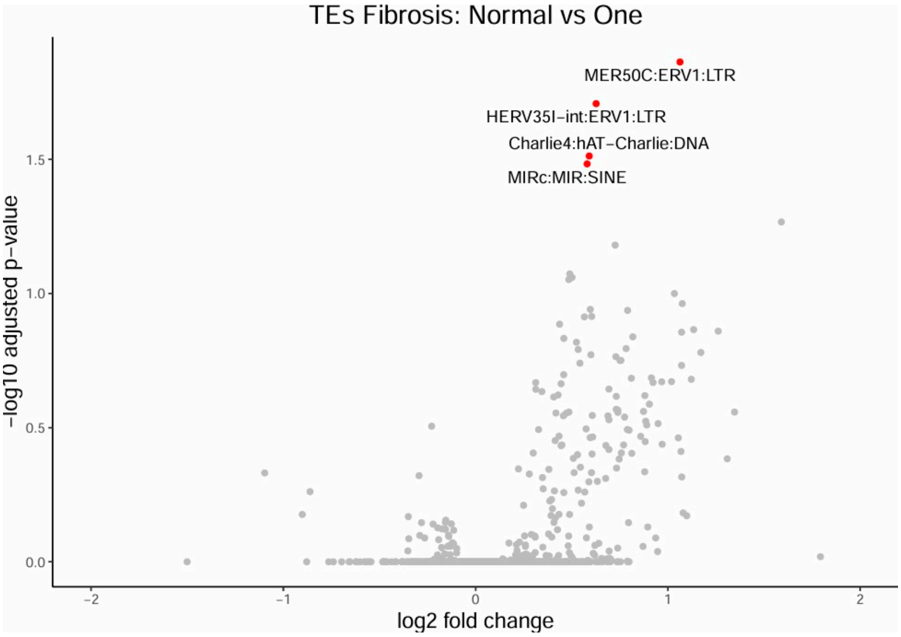

B.

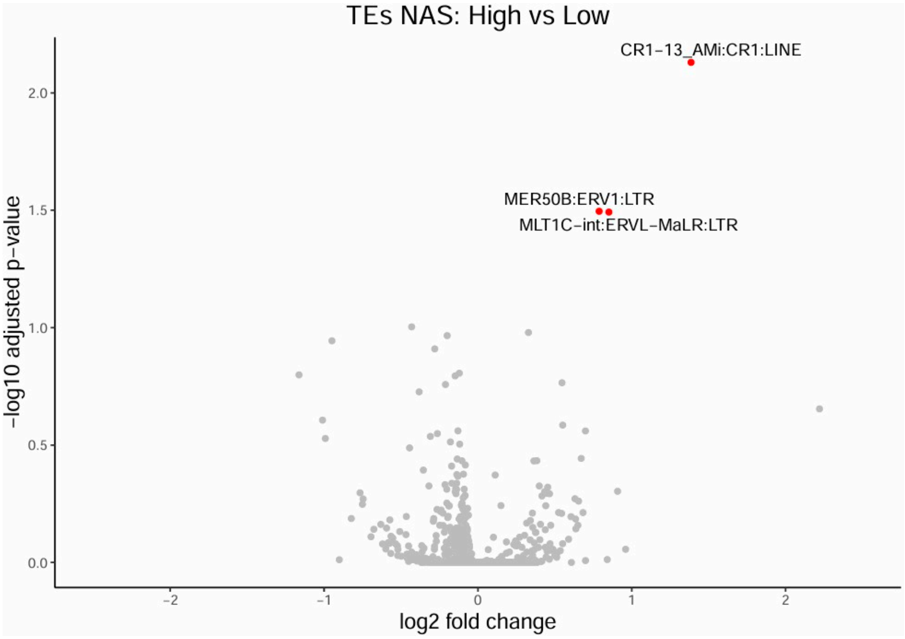

Supplementary Figure S2. Differentially abundant TE transcripts at the subclass level due to A. Fibrosis as determined by Stage normal/0 versus 1 and B. due to Steatosis as determined by NAS (low NAS 0-3 vs high NAS 4-6). Red dots are TE subclasses which Padj<0.05.

Supplementary Tables

Supplementary Table S1. Demographic data in Bariatric Surgery patient cohort stratified by Fibrosis Stage or NAS level.

**Fibrosis**	**0**	**1**	**2**					
**Age (years)**	44.9	46.4	43.7					
**STD**	12.6	14.8	13.2					
**N**	39	25	26					
**SEM**	2.0	3.0	2.6					
**M:F Male %**	30.8	32	26.9					
**NAS**	**0**	**1**	**2**	**3**	**4**	**5**	**6**	**7**
**Age (years)**	47.2	35.91	48.1	49	47.9	443	48.8	37.9
**STD**	10.6	13.7	7.9	13.7	15.1	11.7	16.5	13.8
**N**	13	12	9	11	10	18	10	7
**SEM**	2.9	4.0	2.6	4.1	4.8	2.8	5.2	5.2
**M:F Male %**	23.1	16.7	11.1	36.4	30	44.4	50	14.3

Supplementary Table S2. Selected Differentially abundant genes in Cohort 1 due to Steatosis as determined by NAS (low NAS 0-3, n=42 patients versus high NAS 4-6, n=22 patients). Negative log2 fold change means the gene transcript level is higher in the High NAS group. logFC, log2 fold change; FDR, False Discovery Rate - Multiple testing corrected P value with Bonferroni- Hochberg procedure. P<0.05 in bold.

DNA METHYLATION ASSOCIATED GENES

**Common Name**	**Ensembl Gene Identifier**	**logFC**	**P Value**	**FDR**
DNMT1	ENSG00000130816.17	-0.14	0.15	0.71
DNMT3A	ENSG00000119772.19	-0.07	0.36	0.84
DNMT3B	ENSG00000088305.19	-0.10	0.38	0.84
DNMT3L	ENSG00000142182.9	0.03	0.91	0.99
MAT1A	ENSG00000151224.13	0.19	**0.02**	0.53

MAT2A	ENSG00000168906.13	0.14	0.08	0.64
MECP2	ENSG00000169057.25	0.07	0.18	0.74
TET1	ENSG00000138336.9	-0.08	0.35	0.84
TET2	ENSG00000168769.14	0.00	0.94	0.99
TET3	ENSG00000187605.16	0.04	0.54	0.91
UHRF1	ENSG00000276043.5	-0.27	0.21	0.76

HISTONE MODIFICATION ASSOCIATED GENES

Common Name	Ensembl Gene Identifier	logFC	P Value	FDR
BRD2	ENSG00000204256.14	0.06	0.40	0.85
CHAF1A	ENSG00000167670.16	0.00	0.98	1.00
EED	ENSG00000074266.23	-0.09	0.30	0.81
EHMT1	ENSG00000181090.21	0.06	0.39	0.85
EZH2	ENSG00000106462.12	-0.11	0.19	0.75
G9a/EHMT2	ENSG00000204371.12	0.02	0.88	0.98
HDAC1	ENSG00000116478.12	0.07	0.27	0.79
KAT8	ENSG00000103510.20	0.11	0.10	0.66
LSD1	ENSG00000004487.18	0.00	0.96	0.99
MSL1	ENSG00000188895.12	-0.05	0.39	0.85
MSL2	ENSG00000174579.5	-0.08	0.13	0.69
MSLL3	ENSG00000005302.19	-0.09	0.07	0.63
SETDB1	ENSG00000143379.13	-0.01	0.87	0.98
SUV39H1	ENSG00000101945.17	0.20	0.07	0.63
SUV39H2	ENSG00000152455.16	-0.20	**0.02**	0.54
SUZ12	ENSG00000178691.11	-0.06	0.28	0.80
Tip60/KAT5	ENSG00000172977.13	0.08	0.26	0.78

OTHER TRANSPOSABLE ELEMENT REGULATING GENES

Common Name	Ensembl Gene Identifier	logFC	P Value	FDR
APOBEC3F	ENSG00000128394.17	-0.10	0.33	0.83
APOBEC3G	ENSG00000239713.9	-0.29	**0.03**	0.56
RB1	ENSG00000139687.16	-0.17	**0.02**	0.53
RBL1	ENSG00000080839.12	-0.10	0.16	0.72
RBL2	ENSG00000103479.17	0.09	0.14	0.71

RUNX3	ENSG00000020633.19	-0.21	0.17	0.73
SAMHD1	ENSG00000101347.11	-0.25	0.03	0.55
TRIM28	ENSG00000130726.12	0.17	0.13	0.70
YY1	ENSG00000100811.15	-0.01	0.84	0.98

Supplementary Table S3. Demographic data in MASLD-HCC study cohort (mean ± SEM).

		MASLD				
	**Normal Liver (n=27)**	**Overall (n=45)**	**Simple Steatosis (n=21)**	**MASH (n=11)**	**Cirrhosis (n=13)**	**MASLD-Associated HCC (n=14)**
**Age (years)**	57 ± 2	59 ± 2	55 ± 3	62 ± 4	63 ± 3	70 ± 2 [1,2,3]
**M:F**<br>**Male %**	15:12<br>56	26:19<br>58	14:7<br>67	5:6<br>46	7:6<br>54	12:2<br>86
**BMI (kg/m2)**	24.6 ± 0.4	27.4 ± 0.3 [4]	27.9 ± 0.5 [4]	27.5 ± 0.7 [5]	26.6 ± 0.6 [6]	26.7 ± 0.4 [7]

1. P= 0.0002 compared with Normal Liver
2. P=0.006 compared with MASLD Overall
3. P=0.002 compared with Simple Steatosis
4. P<0.0001 compared with Normal Liver
5. P=0.0003 compared with Normal Liver
6. P=0.006 compared with Normal Liver
7. P=0.0016 compared with Normal Liver

Supplementary Table S4. Distributions of steatosis grades, non-alcoholic fatty fiver activity scores and fibrosis stages in 45 patients with MAFLD in MASLD-HCC study cohort.

Steatosis Grade		
	1	n=10, 22%
	2	n=25, 56%
	3	n=10, 22%
Non-alcoholic Fatty Liver Activity Score		
	1-3	n=28, 62%
	≥5	n=17, 38%
Fibrosis Grade		
	0	n=24, 53%
	1	n=3, 7%
	2	n=1, 2%
	3	n=4, 9%
	4	n=13, 29%

Supplementary Table S5. Correlations between fibrosis grade and (i) steatosis grade and (ii) non-alcoholic fatty liver activity score in 45 patients with MASLD in MASLD-HCC study cohort.

	**r**	**P**
**Fibrosis Grade**		
**Steatosis Grade**	0.25	0.10
**Non-alcoholic Fatty Liver Activity Score**	**0.44**	**0.003**

Supplementary Table S6. Study parameters in relation to gender (median, range) in MASLD-HCC study cohort.

	**MALE**	**FEMALE**
**Alu**	(n=25)<br>0.007412 (0 – 107100)	(n=24)<br>0.005198 (0 – 0.154500)
**L1**	(n=25)<br>0.000168 (0 – 0.003606)	(n=24)<br>0.000328 (0 – 0.004378)
**L1 METHYLATION**	(n=24)<br>44.0 (7.2 – 96.1)	(n=14)<br>53.0 (8.9 – 85.2)

Supplementary Table S7. Demographic data in ARLD patients (cohort 3)

	AGE	GENDER	BMI	ALCOHOL (G/DAY)
	n=25	n=25	n=25	n=21
**M:F**		16:9		
**MALE %**		64%		
**MEAN**	48.6		26.2	255
**SD**	11		4	154.5
**MEDIAN**	46		26	240
**RANGE**	32-73		18.6-33.8	100-750
